# Supplementary material for: Meta-analysis of the severe acute respiratory syndrome coronavirus 2 serial intervals and the impact of parameter uncertainty on the coronavirus disease 2019 reproduction number
Source: Stat Methods Med Res. 2021 Dec 21;31(9):1686–703. doi: 10.1177/09622802211065159 (PMC9465543; doi:10.1177/09622802211065159)
Supplement: sj-pdf-1-smm-10.1177_09622802211065159 - Supplemental material for Meta-analysis of the severe acute respiratory syndrome coronavirus 2 serial intervals and the impact of parameter uncertainty on the coronavirus disease 2019 reproduction number [file sj-pdf-1-smm-10.1177_09622802211065159.pdf]

Meta-analysis of the SARS-CoV-2 serial interval and the impact of parameter uncertainty on the COVID-19 reproduction number - Supplementary 1

Robert Challen[1,2,3]; Ellen Brooks-Pollock[3,4]; Krasimira Tsaneva-Atanasova[1,5,6]; Leon Danon[4,5,6,7]

1) EPSRC Centre for Predictive Modelling in Healthcare, University of Exeter, Exeter, Devon, UK.
2) Somerset NHS Foundation Trust, Taunton, Somerset, UK.
3) Joint Universities Pandemic and Epidemiological Research (JUNIPER) consortium.
4) Bristol Medical School, Population Health Sciences, University of Bristol, Bristol, UK.
5) The Alan Turing Institute, British Library, 96 Euston Rd, London NW1 2DB, UK.
6) Data Science Institute, College of Engineering, Mathematics and Physical Sciences, University of Exeter, Exeter, UK.
7) Department of Engineering Mathematics, University of Bristol, UK.

Supporting tables and figures

Supplemental table 1: An algorithm for resampling representative serial intervals based on paramterised distributions and raw data from published studies

The detail of the re-sampling algorithm that is used to combine estimates from a range of different studies is given below. The rationale for the definition of the sampling distributions is covered in more detail in Supplementary Materials 2. Teh algorithm combines raw data from original studies, with synthetic data from reasmpling the uncertain parameterised distributions provided in the literature. The combination of these is then used to compare different maximum likelihood parametric distributions fitting all the data, and later as a target for optimising the generation interval distribution when combined with a dataset of incubation period estimates.

INPUTS:

parameterised serial interval distributions:
list of parameterised serial interval distribution results from literature sources
comprising:
source, the study the estimates come from
distribution, type of estimated serial interval distribution
central estimate of mean,
lower CI of mean, if available
upper CI of mean, if available
central estimate of sd,
lower CI of sd, if available
upper CI of sd, if available
sample size

raw serial interval observations:
raw data of empirical serial interval distributions from literature sources
comprising:

        source,
        set(***observed serial interval***)

OUTPUT:

output serial interval observations:
    a set of 100 bootstrap replicates containing real and synthetic serial intervals observations
    comprising:
        iteration,
        set(***simulated serial interval***)

ALGORITHM:

for each ***source*** in ***raw serial interval observations***
    define ***sample size*** as the count of ***observed serial interval***
    for ***iteration*** in ***1 to 100***
        define set(***samples***) as random re-samples with replacement from ***observed serial interval***
        add (***iteration***, set(***samples***)) to ***output serial interval observations***
for each ***source*** in ***parameterised serial interval distributions***
    — we are recreating a set of sampling distributions $\mathcal{X}(\nu, \phi)$
    — where $\mathcal{X}$ as ***distribution*** from study ***source*** (e.g. gamma, log-normal, normal)
    — ν is a distribution of means compatible with study ***source***
    — and ϕ is a distribution of std devns compatible with study ***source***
    — ν is assumed to be a truncated normal distribution
    — and is the sample distribution of the mean
    define μ_{mean} as ***central estimate of mean***
    define σ_{mean} as ***(upper CI of mean-lower CI of mean)/3.96*** or ***zero*** if CI not given
    define $\nu \sim \mathcal{N}(\mu_{mean}, \sigma_{mean})$ truncated between ***lower CI of mean*** and ***upper CI of mean***
    — ϕ is a Nakagami distributed quantity variously parameterised
    — depending on whether we have known confidence limits
    — this is the sample distribution of the standard deviation
    define μ_{sd} as ***central estimate of sd***
    define N as ***sample size***
    if ***lower CI of sd*** and ***upper CI of sd*** are defined
        — if we know confidence limits and mean of SD we assume it is Nakagami distributed
        — with Ω parameter as σ^2
        fit $\phi \sim \text{Nakagami}(m_{sd}, \sigma^2)$ to ***lower CI of sd*** and ***upper CI of sd***
    else
        — if we know only know mean of SD (and sample size) we assume ϕ has a different parameterisation
        define $\phi \sim \text{Nakagami}(\frac{N-1}{2}, \sigma^2)$
    for ***iteration*** in ***1 to 100***
        define μ_{sample} as random sample from ν
        define σ_{sample} as random sample from ϕ
        define ***sampling distribution*** $\sim \mathcal{X}(\mu_{sample}, \sigma_{sample})$; converting parameters as necessary
        define set(***samples***) as N random samples from ***sampling distribution***
        add (***iteration***, set(***samples***)) to ***output serial interval observations***
return ***output serial interval observations***

Supplemental table 2: The NHS trusts in the CHESS dataset who report all admissions

The CHESS dataset includes a wide range of contributing hospitals with very varied data quality of submission. Only some hospitals continue to update their data, to include updates to patients as they are discharged or die, and only some submit data on all inpatients not just those who go to ITU. While investigating the delays to various events in the hospital stay we focussed on a subset of contributing hospitals that last updated records within 21 days of the date of analysis, and for which had a maximum of 20% of their cases had unknown or incomplete dates. This included the following contributing trusts and within these we excluded patients that had their COVID-19 diagnosis made more than 10 days after admission.

Trust	Patients
BARKING, HAVERING AND REDBRIDGE UNIVERSITY HOSPITALS NHS TRUST	1056
BLACKPOOL TEACHING HOSPITALS NHS FOUNDATION TRUST	558
CHESTERFIELD ROYAL HOSPITAL NHS FOUNDATION TRUST	440
GREAT WESTERN HOSPITALS NHS FOUNDATION TRUST	398
LIVERPOOL HEART AND CHEST HOSPITAL NHS FOUNDATION TRUST	523
NORTH CUMBRIA INTEGRATED CARE NHS FOUNDATION TRUST	360
NORTH WEST ANGLIA NHS FOUNDATION TRUST	758
ROYAL BERKSHIRE NHS FOUNDATION TRUST	629
SOUTH TEES HOSPITALS NHS FOUNDATION TRUST	85
TAMESIDE HOSPITAL NHS FOUNDATION TRUST	686
THE NEWCASTLE UPON TYNE HOSPITALS NHS FOUNDATION TRUST	514
THE QUEEN ELIZABETH HOSPITAL, KING'S LYNN, NHS FOUNDATION TRUST	401
UNIVERSITY HOSPITAL SOUTHAMPTON NHS FOUNDATION TRUST	571
WALSALL HEALTHCARE NHS TRUST	700
WEST HERTFORDSHIRE HOSPITALS NHS TRUST	688
WORCESTERSHIRE ACUTE HOSPITALS NHS TRUST	787
YEOVIL DISTRICT HOSPITAL NHS FOUNDATION TRUST	80
YORK TEACHING HOSPITAL NHS FOUNDATION TRUST	668

Supplemental figure 1: Forest plot for serial interval studies for the mean of the serial interval, using the normal mixture random effect model, and from studies identified in the literature which give confidence intervals

Assessing the serial interval distribution using a random effects meta-analysis of the studies that report a confidence limit for the mean has the benefit of using a standardised methodology but does not describe the distributional nature of the serial interval.

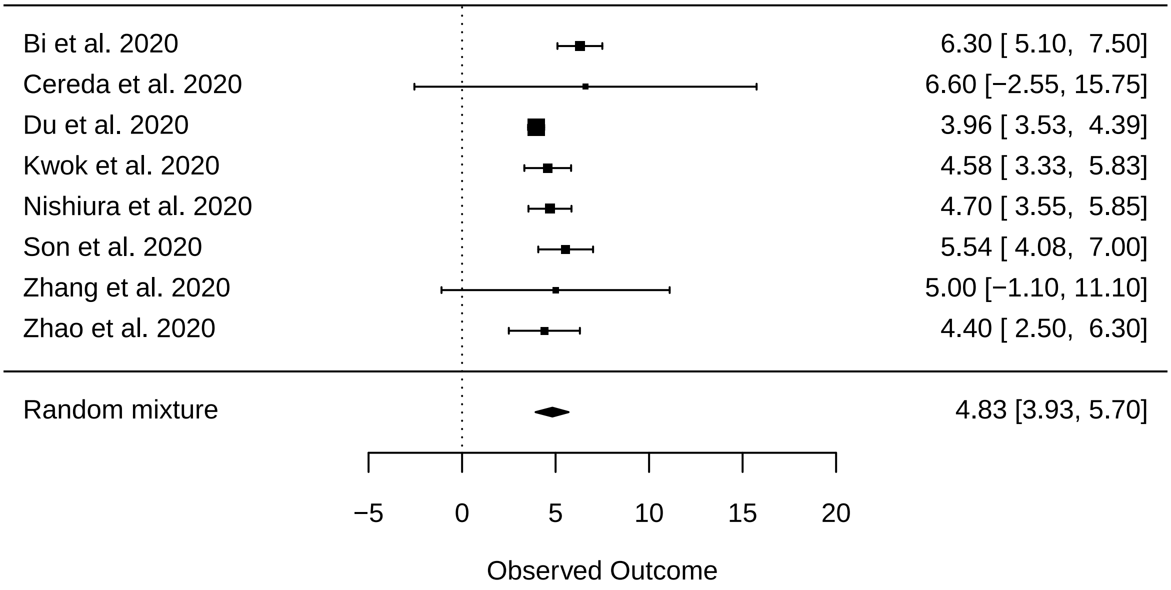

Supplemental table 3: Parametererised serial interval distributions from resampling the literature. Gamma and weibull estimates are from data truncated at zero. AIC estimates are not comparable to those for Normal distribution which is fitting all data, including negative serial intervals, and hence has a lower mean.

Maximum likelihood parameterised distributions fitted to the resampled serial interval data demonstrate variable quality of fits, as the serial interval data has a substantial component which is negative and therefore fitting continuous distributions with support in the positive real numbers requires truncating the data at zero as seen in Figure 2 in the main paper.

Distribution	AIC	N	Parameter / Moment	Mean ± SD (95% CI)
gamma	5081.1	92380	scale	3.86 ± 0.44 (3.31; 4.83)
			shape	1.50 ± 0.20 (1.09; 1.75)
			mean	5.72 ± 0.38 (5.08; 6.51)
			sd	4.69 ± 0.27 (4.28; 5.30)
normal	6106.4	101178	mean	4.87 ± 0.37 (4.24; 5.52)
			sd	4.79 ± 0.26 (4.45; 5.42)
			mean	4.87 ± 0.37 (4.24; 5.52)
			sd	4.79 ± 0.26 (4.45; 5.42)
weibull	5056.9	92380	scale	6.19 ± 0.48 (5.32; 7.09)
			shape	1.33 ± 0.10 (1.13; 1.46)
			mean	5.70 ± 0.38 (5.06; 6.49)
			sd	4.34 ± 0.27 (3.96; 4.91)

Supplemental table 4: Parametererised serial interval distributions from FF100. Gamma and weibull estimates are from data truncated at zero. AIC estimates are not comparable to those for Normal distribution which is fitting all data, including negative serial intervals, and hence has a lower mean.

The direct estimation of the serial interval from the FF100 data does not produce an estimatate that is comparable with other international data sets.

Distribution	AIC	N	Parameter / Moment	Mean ± SD (95% CI)
gamma	182	43	shape	2.77 ± 0.49 (2.01; 3.78)
			scale	1.30 ± 0.24 (0.91; 1.77)
			mean	3.50 ± 0.33 (2.84; 4.14)
			sd	2.13 ± 0.25 (1.59; 2.61)
normal	251	50	mean	2.81 ± 0.41 (2.03; 3.54)
			sd	2.74 ± 0.34 (2.16; 3.41)
			mean	2.81 ± 0.41 (2.03; 3.54)
			sd	2.74 ± 0.34 (2.16; 3.41)
weibull	183	43	shape	1.75 ± 0.18 (1.43; 2.17)
			scale	3.94 ± 0.37 (3.21; 4.67)
			mean	3.52 ± 0.32 (2.85; 4.14)
			sd	2.08 ± 0.23 (1.62; 2.51)

Supplemental table 5: Distribution details for estimated incubation period distributions reconstructed from Open COVID-19 Data Working Group and from FF100 data

Detail of the distribution parameterisation of the incubation period with the 3 investigated distributions on the 2 data sets, shows that in the larger Open COVID-19 Data working group data set the best fit is obtained with a log-normal distribution.

N	Source	AIC	Distribution	Parameter / Moment	Mean ± SD (95% CI)
33	FF100	62.1	gamma	shape	1.71 ± 4.14 (0.35; 4.81)
				scale	1.68 ± 0.85 (0.45; 3.71)
				mean	1.82 ± 0.35 (1.10; 2.50)
				sd	1.67 ± 0.42 (0.86; 2.42)
			weibull	shape	1.23 ± 0.39 (1.00; 1.99)
				scale	1.94 ± 0.37 (1.28; 2.62)
				mean	1.85 ± 0.32 (1.28; 2.51)
				sd	1.57 ± 0.33 (0.93; 2.20)
		63.5	log-normal	meanlog	0.25 ± 0.34 (-0.59; 0.68)
				sdlog	0.85 ± 0.24 (0.42; 1.36)
				mean	1.94 ± 0.34 (1.26; 2.60)
				sd	2.11 ± 0.93 (0.88; 4.43)
1062	Open COVID-19 Data Working Group	5157.2	log-normal	meanlog	1.27 ± 0.03 (1.21; 1.32)
				sdlog	0.87 ± 0.01 (0.84; 0.89)
				mean	5.19 ± 0.15 (4.90; 5.46)
				sd	5.49 ± 0.24 (5.05; 5.92)
		5191.7	gamma	shape	1.55 ± 0.05 (1.46; 1.65)
				scale	3.26 ± 0.15 (2.96; 3.52)
				mean	5.06 ± 0.14 (4.80; 5.29)
				sd	4.06 ± 0.14 (3.78; 4.30)
		5216.6	weibull	shape	1.25 ± 0.03 (1.20; 1.30)
				scale	5.45 ± 0.15 (5.17; 5.73)
				mean	5.08 ± 0.14 (4.82; 5.32)
				sd	4.09 ± 0.14 (3.80; 4.33)

Supplemental figure 2: Graphical goodness of fit for parameterised incubation period distributions fitted to the Be Outbreak Prepared dataset

Further investigation of the fit of the log-normal incubation period demonstrates good alignment of the model fits in all models to the early part of the distribution but with some loss of fit towards the tails, due to a number of censored entries starting after day 28. These data are generally irrelevant given the incubation period distribution is largely only subsequently used in a discretised form up to a limited number of days. This poorly fitting tail could influence the upper confidence limit of the incubation period which may be slightly longer than anticipated.

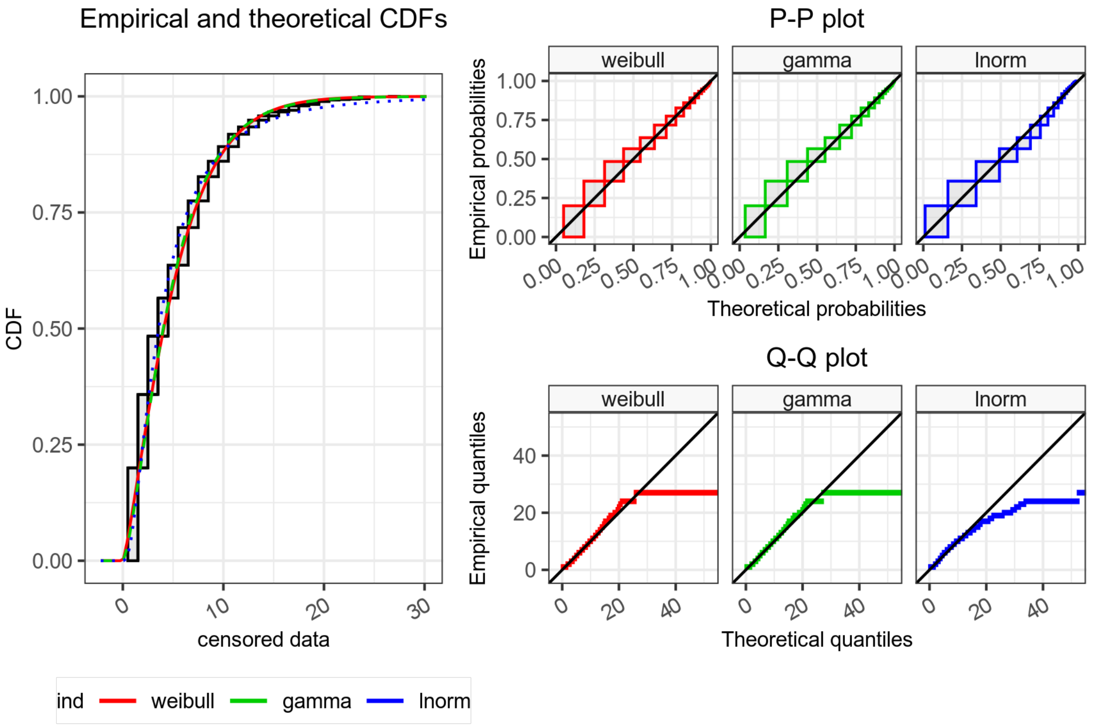

Supplemental figure 3: The distribution of the parameters of the fitted generation interval estimates

The range of generation distributions resulting from optimising the different combinations of bootstrapped serial interval and incubation periods shows a spread of possible generation interval distributions, but mean and standard deviations are not wholly independent as seen in panel A, with a tendency to increasing SD with increasing mean. The shape and scale parameterisation shows a close reciprocal relationship between them due in some part to the constraints placed on the mean of the distributions.

The finding there the generation time mean and standard deviation distributions are not independent raises a question about whether sampling from uncertainly specified distributions can ever produce a realistic sample of generation time distributions for subsequent use in analysis. For example in estimating R_t using the EpiEstim package uncertain parameterised distributions are sampled using independent truncated normal distributions on the mean and standard deviation and their associated confidence intervals. This will tend to broaden the range of considered distributions beyond that seen here, and hence increase uncertainty. It is possible that re-sampling using independent truncated normal distributed means and standard deviation could also bias estimates of R_t but it is not obvious in which direction or by how much. This is an area for future investigation.

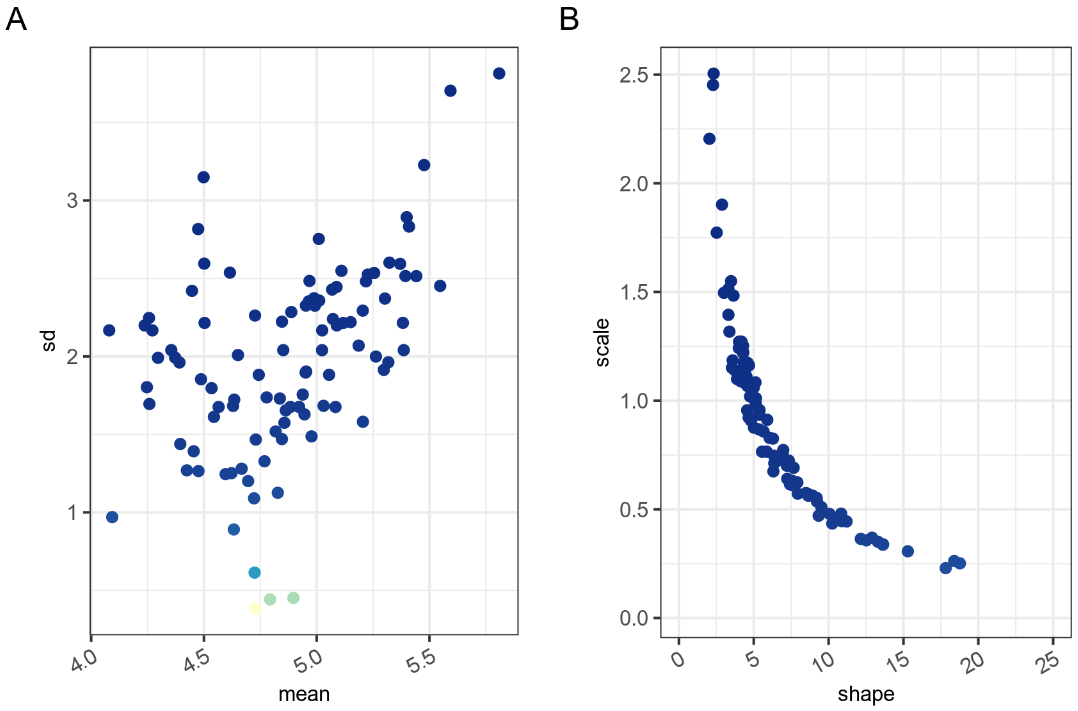

Supplemental table 6: Time delay distributions estimated from CHESS data set, for both transitions from disease onset to case, admission or death, and presumed infection and case, admission or death

The combination of incubation period and delay from symptom onset to positive test, admission or death are graphically displayed in Figure 7. Here the details of these parameterisations are provided. In all cases the best fit is obtained with a log-normal distribution. The infection to test distribution is used in the de-convolution analysis presented in the main paper.

Transition	AIC	Distribution	Parameter	Mean ± SD (95% CI)
infection to admission	5262.3	log-normal	meanlog	1.61 ± 0.04 (1.51; 1.67)
			sdlog	0.93 ± 0.02 (0.88; 0.97)
			mean	7.70 ± 0.26 (7.22; 8.08)
			sd	9.05 ± 0.52 (7.93; 10.00)
	5361.7	gamma	scale	5.80 ± 0.40 (5.05; 6.57)
			shape	1.32 ± 0.07 (1.19; 1.44)
			mean	7.64 ± 0.26 (7.13; 8.07)
			sd	6.65 ± 0.32 (6.04; 7.36)
	5388.0	weibull	scale	7.93 ± 0.25 (7.34; 8.34)
			shape	1.09 ± 0.04 (1.02; 1.15)
			mean	7.68 ± 0.26 (7.16; 8.10)
			sd	7.05 ± 0.37 (6.30; 7.74)
infection to death	6282.2	log-normal	meanlog	2.58 ± 0.02 (2.55; 2.61)
			sdlog	0.62 ± 0.01 (0.60; 0.65)
			mean	16.04 ± 0.28 (15.57; 16.56)
			sd	11.08 ± 0.38 (10.31; 11.85)
	6319.9	gamma	scale	5.77 ± 0.28 (5.25; 6.34)
			shape	2.77 ± 0.11 (2.58; 2.99)
			mean	15.98 ± 0.29 (15.49; 16.55)
			sd	9.60 ± 0.30 (9.05; 10.24)
	6406.6	weibull	scale	17.97 ± 0.32 (17.43; 18.56)
			shape	1.62 ± 0.04 (1.55; 1.71)
			mean	16.09 ± 0.30 (15.59; 16.69)
			sd	10.19 ± 0.37 (9.43; 10.97)
infection to test	4936.5	gamma	scale	3.68 ± 0.18 (3.35; 4.07)
			shape	1.75 ± 0.08 (1.58; 1.90)
			mean	6.43 ± 0.15 (6.11; 6.69)
			sd	4.86 ± 0.15 (4.56; 5.18)
	4953.3	weibull	scale	7.06 ± 0.17 (6.68; 7.36)
			shape	1.36 ± 0.04 (1.28; 1.43)
			mean	6.46 ± 0.15 (6.14; 6.72)
			sd	4.80 ± 0.15 (4.51; 5.12)
	4969.0	log-normal	meanlog	1.55 ± 0.03 (1.48; 1.60)
			sdlog	0.85 ± 0.03 (0.80; 0.89)
			mean	6.73 ± 0.17 (6.41; 7.03)
			sd	6.89 ± 0.35 (6.30; 7.57)
