# Supplementary material for: Meta-analysis of the severe acute respiratory syndrome coronavirus 2 serial intervals and the impact of parameter uncertainty on the coronavirus disease 2019 reproduction number
Source: Stat Methods Med Res. 2021 Dec 21;31(9):1686–703. doi: 10.1177/09622802211065159 (PMC9465543; doi:10.1177/09622802211065159)
Supplement: sj-pdf-2-smm-10.1177_09622802211065159 - Supplemental material for Meta-analysis of the severe acute respiratory syndrome coronavirus 2 serial intervals and the impact of parameter uncertainty on the coronavirus disease 2019 reproduction number [file sj-pdf-2-smm-10.1177_09622802211065159.pdf]

Meta-analysis of the SARS-CoV-2 serial interval and the impact of parameter uncertainty on the COVID-19 reproduction number - Supplementary 2

Robert Challen[1,2,3]; Ellen Brooks-Pollock[3,4]; Krasimira Tsaneva-Atanasova[1,5,6]; Leon Danon[4,5,6,7]

1) EPSRC Centre for Predictive Modelling in Healthcare, University of Exeter, Exeter, Devon, UK.
2) Somerset NHS Foundation Trust, Taunton, Somerset, UK.
3) Joint Universities Pandemic and Epidemiological Research (JUNIPER) consortium.
4) Bristol Medical School, Population Health Sciences, University of Bristol, Bristol, UK.
5) The Alan Turing Institute, British Library, 96 Euston Rd, London NW1 2DB, UK.
6) Data Science Institute, College of Engineering, Mathematics and Physical Sciences, University of Exeter, Exeter, UK.
7) Department of Engineering Mathematics, University of Bristol, UK.

Sampling from uncertain distributions

Introduction

This is a brief note to explain the approach taken to producing samples from results given as parameterised distributions cited in the literature. The purpose for this is in combining multiple studies which report a quantity (e.g. the serial interval of an infection) as modelled by a parameterised statistical distribution (e.g. a Gamma distribution), with a central estimate of the mean, a central estimate of the standard deviation, and typically confidence limits on both of those quantities (or credible intervals if the distribution was estimated using a Bayesian framework). Combining such results into a single estimate through meta-analysis does not fit within the standard approaches, as these generally assume a normally distributed single dimensional effect, and whilst this is probably valid for the means of the parameterised distributions to be treated in this way, it is not necessarily a valid assumption for the parameter defining the spread (i.e. the standard deviation).

The challenge in combining such distributions is essentially that of estimating the mixture of all possible distributions that are compatible with the results published in all the studies. The resulting mixture distribution may then be further analysed in a range of ways.

For example an additional important capability for us is the ability to combine studies that present results as a parameterised distribution, with other studies where only empirical estimates are made on the quantities of interest, and the raw data is available. In this case generating representative samples from the original report is important, so that parameterised results can be combined with empirical results. This approach is akin to parametric bootstrapping, but where the bootstrapping is performed not on data but on the uncertain estimate of the parameterised distribution.

The key step of this re-sampling is the conversion of an uncertain parameterised distribution into a representative set of precisely specified distributions that can in turn be sampled. The set of studies described in Table 1 is a typical example of the kind of information analysed with this approach.

The specific problem of generating a set of representative set of precisely specified parameterised distributions from an uncertainly specified result is somewhat similar to that of sampling parameter values within a Bayesian framework where the mean and standard deviation of a parameter distribution are themselves

specified by prior distributions. In this scenario however the choice of distribution for the mean and spread parameter (usually variance) as hyper-parameters, can be assumed. Because of the Central Limit theorem, a sensible choice for the prior of the mean is a Gaussian distribution, but the spread parameter typically has support between zero and infinity, and often weakly informed priors chosen for this, from either uniform distributions or half-t family (including the Cauchy distribution) (Gelman 2006)[1].

In our situation, we are doing the reverse, and given a mean and standard deviation, and confidence intervals for each, but no knowledge of the distributions of these quantities, the challenge is to produce a set of sampling distributions that accurately reflect the study definition.

The sampling distribution of the mean

To do this we need to make some assumptions about the nature of the sampling distribution of the mean. Fortunately this is rather simple, as a key finding of the central limit theorem, as regardless of the underlying distribution, as the number of samples of a distribution increases the sampling distribution of the mean (E_n) is a Gaussian where $\bar{\mu}$ is the central estimate of the mean[2]:

$$E_n \sim \mathcal{N}(\bar{\mu}, \frac{\bar{\sigma}}{\sqrt{n}})$$

Knowing that the sampling distribution of the mean is a Gaussian, we can use this assumption to estimate the $\frac{\bar{\sigma}}{\sqrt{n}}$ quantity, which is the standard deviation of the sampling distribution of the mean, from the confidence intervals, giving us a fully specified sampling distribution of the mean.

$$E_n \sim \mathcal{N}(\bar{\mu}, \frac{CI_{\mu,upper} - CI_{\mu,lower}}{\mathcal{N}_{upper} - \mathcal{N}_{lower}})$$

The sampling distribution of the variance and standard deviation

A normally distributed variable x with an expected value μ and a standard deviation σ is sampled n times. the set of x_n observations has a mean of $\overline{x}$ and observed variance of S_n^2, the sampling distribution of the variance can be shown to be a Chi-squared distribution[2] with $n-1$ degrees of freedom. Given that the Chi-squared distribution is a particular form of a Gamma distribution (here parameterised with shape, α and rate, β) and given the definition of the Nagakami-m distribution[3], the following holds:

$$\begin{aligned}
(n-1)S_n^2/\sigma^2 = \frac{1}{\sigma^2}\sum_i (x_i - \overline{x})^2 \sim \tilde{\chi}_{n-1}^2 \\
\tilde{\chi}_{n-1}^2 = \mathrm{Gamma}\Big(\frac{n-1}{2}, \frac{1}{2}\Big) \\
X \sim \mathrm{Gamma}(\alpha, \beta) \implies yX \sim \mathrm{Gamma}(\alpha, \beta/y) \\
S_n^2 = \frac{\sigma^2}{n-1}\left(\frac{(n-1)S_n^2}{\sigma^2}\right) \sim \mathrm{Gamma}\left(\frac{n-1}{2}, \frac{n-1}{2\sigma^2}\right) \\
m = \kappa = \alpha = \frac{n-1}{2} \\
\Omega = \kappa\theta = \frac{\alpha}{\beta} = \sigma^2 \\
X \sim \mathrm{Gamma}(\alpha, \beta) \implies \sqrt{X} \sim \mathrm{Nakagami}(m, \Omega) \\
S_n \sim \mathrm{Nakagami}(\frac{n-1}{2}, \sigma^2)
\end{aligned}$$

With no information about the nature of the underlying distribution this expression is a bounding limit on the sampling distribution of the standard deviation, and we use this in the situation where the central estimate of the standard deviation is given, alongside the sample size, but with no other information. However this estimate is unreliable in the situation where there are small numbers or there is kurtosis in the distribution[4], and could lead to a broader range of samples than would be compatible with the reported results when these are Gamma, or Log-normally distributed.

In O'Neill (2014)[5] the asymptotic sampling distribution of the variance is explored with respect to the kurtosis of the underlying distribution, and this modifies the degrees of freedom applied to the Chi-squared distribution above, to the following expression, where κ is the kurtosis of the underlying distribution (this is their result 14).

$$
\begin{aligned}
DF_n S_n^2/\sigma^2 &\sim \tilde{\chi}^2_{DF_n} \\
DF_n &= \frac{2n}{\kappa - (n-3)/(n-1)} \\
S_n^2 = \frac{\sigma^2}{DF_n}\left(\frac{(DF_n)S_n^2}{\sigma^2}\right) &\sim \text{Gamma}\left(\frac{DF_n}{2}, \frac{DF_n}{2\sigma^2}\right) \\
S_n &\sim \text{Nakagami}(\frac{DF_n}{2}, \sigma^2)
\end{aligned}
$$

Information about the kurtosis of the underlying distribution is available from the confidence limits on the standard deviations quoted in source studies and a closed form expression for these is given in O'Neill (2014)[4]. This involves the population size from which the sample is taken which is information we do not generally have. With both confidence intervals, it would be possible to eliminate the unknown population size (or we could reasonably assume it is very much larger than our sample size), but it is also possible to estimate the associated Nakagami distribution numerically from the confidence intervals and central estimate of the standard deviation (σ) from the expression above. These again describe bounding distributions for the sampling distribution of the standard deviation.

Generating samples from uncertain distributions

The main purpose of this approach is to generate a representative sample set from uncertainly specified parameterised distributions such that they can be combined. To test this we investigate a list of published studies that give estimates of the serial interval of SARS-CoV-2 as a parameterised distribution (see Table S2.1).

Table S2.1: A set of uncertainly specified parameterised distributions extracted from the SARS-CoV-2 literature.

source	dist	N	param	summary
Bi et al. 2020	gamma	48	mean	6.30, (95% CI 5.20–7.60)
			sd	4.20, (95% CI 3.10–5.30)
Cereda et al. 2020	gamma	90	mean	6.60, (95% CI 0.70–19.00)
			sd	4.88, (95% CI NA–NA)
Du et al. 2020	norm	468	mean	3.96, (95% CI 3.53–4.39)
			sd	4.75, (95% CI 4.46–5.07)
Kwok et al. 2020	lnorm	26	mean	4.58, (95% CI 3.35–5.85)
			sd	3.28, (95% CI 2.18–4.01)
Nishiura et al. 2020	lnorm	28	mean	4.70, (95% CI 3.70–6.00)
			sd	2.90, (95% CI 1.90–4.90)
Son et al. 2020	gamma	28	mean	5.54, (95% CI 4.08–7.01)
			sd	3.90, (95% CI 2.47–5.32)
Zhang et al. 2020	gamma	28	mean	5.00, (95% CI 0.80–13.00)
			sd	3.22, (95% CI NA–NA)
Zhao et al. 2020	gamma	21	mean	4.40, (95% CI 2.90–6.70)
			sd	3.00, (95% CI 1.80–5.80)

Using the methodology described above, for each of these studies a sampling distribution for the mean and standard deviation is estimated. This is used to generate 1000 representative parameterised distributions for

each study. From these 1000 distributions, 1000 random samples are taken representing 1,000,000 generated samples per study. With all studies taken together and with equally weighting, the combined sample has a mean and SD of 5.32 ± 4.34, (95% CI -0.03–15.99) however in reality we would take a number of samples proportional to the study size when combining.

More relevant though is comparing the distribution of means and standard deviations recovered from the re-sampling process. In this case keeping 1000 sample from each of the 1000 inferred distributions from each study separate, and summarizing the samples shows us how well the distribution sampling is performing. In the following figure, for the Bi et al. 2020 study, we see the sampled mean and standard deviation in each of the 1000 precisely specified distributions, compared to the quoted central estimates (solid red) and confidence intervals (dashed lines) in Panel A, and in panel B the associate shape and rate parameters.

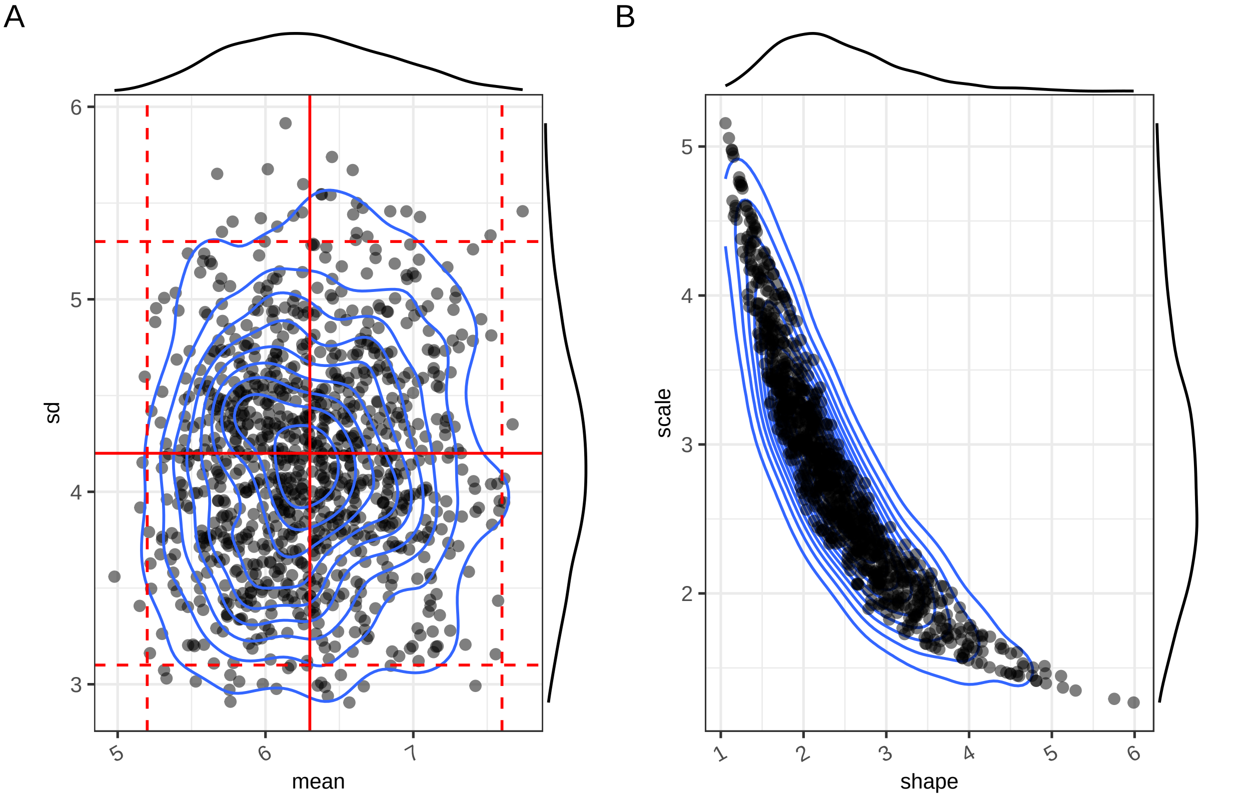

Figure S2.1: Panel A shows the first 2 moments of a set of precisely specified parameterised distributions compatible with the findings of Bi et al. 2020. Central estimates and confidence intervals from that study are marked in red lines in panel A. Panel B is the equivalent distributions expressed in shape and scale parameters of the Gamma distribution.

Combining the summaries from the Figure S2.1 above, allows us to reconstruct the uncertainty in the mean and standard deviation in our sampled data, and reconstruct central estimates and confidence intervals, for each source, which are shown in Table S2.2. These compare well with the originally reported values from the papers, where the numbers of cases is sufficiently large, or the reported confidence intervals are not excessively wide. It is less accurate where the very small numbers in some of the studies leads to wide confidence intervals, for example Zhang et al. 2020. In such cases the ability to replicate the exact shape is arguably less important for our intended purpose as such small studies will be relatively down-weighted during meta-analysis.

Table S2.2: The comparison between original uncertain distributions, and the result of aggregating samples generated using the procedure described in this paper. A good agreement is shown when source distributions are reasonably well constrained to begin with

source	dist	N	param	type	summary
Bi et al. 2020	gamma	48	mean	original	6.30, (95% CI 5.20–7.60)
				sampled	6.29 ± 0.53, (95% CI 5.35–7.33)
			sd	original	4.20, (95% CI 3.10–5.30)
				sampled	4.16 ± 0.56, (95% CI 3.13–5.31)
Cereda et al. 2020	gamma	90	mean	original	6.60, (95% CI 0.70–19.00)
				sampled	7.41 ± 3.85, (95% CI 1.17–15.11)
			sd	original	4.88, (95% CI NA–NA)
				sampled	4.86 ± 0.45, (95% CI 4.07–5.70)
Du et al. 2020	norm	468	mean	original	3.96, (95% CI 3.53–4.39)
				sampled	3.96 ± 0.23, (95% CI 3.52–4.42)
			sd	original	4.75, (95% CI 4.46–5.07)
				sampled	4.75 ± 0.18, (95% CI 4.41–5.12)
Kwok et al. 2020	lnorm	26	mean	original	4.58, (95% CI 3.35–5.85)
				sampled	4.62 ± 0.55, (95% CI 3.56–5.71)
			sd	original	3.28, (95% CI 2.18–4.01)
				sampled	3.22 ± 0.50, (95% CI 2.34–4.25)
Nishiura et al. 2020	lnorm	28	mean	original	4.70, (95% CI 3.70–6.00)
				sampled	4.73 ± 0.51, (95% CI 3.82–5.74)
			sd	original	2.90, (95% CI 1.90–4.90)
				sampled	2.85 ± 0.77, (95% CI 1.83–4.61)
Son et al. 2020	gamma	28	mean	original	5.54, (95% CI 4.08–7.01)
				sampled	5.52 ± 0.67, (95% CI 4.25–6.78)
			sd	original	3.90, (95% CI 2.47–5.32)
				sampled	3.84 ± 0.71, (95% CI 2.53–5.23)
Zhang et al. 2020	gamma	28	mean	original	5.00, (95% CI 0.80–13.00)
				sampled	5.51 ± 2.66, (95% CI 1.17–11.16)
			sd	original	3.22, (95% CI NA–NA)
				sampled	3.17 ± 0.45, (95% CI 2.36–4.07)
Zhao et al. 2020	gamma	21	mean	original	4.40, (95% CI 2.90–6.70)
				sampled	4.49 ± 0.84, (95% CI 3.09–6.21)
			sd	original	3.00, (95% CI 1.80–5.80)
				sampled	2.89 ± 0.97, (95% CI 1.75–5.29)

Conclusion

We have presented a short summary on the method we use to generate samples from uncertainly specified parameterised distributions. We have demonstrated it is able to produce both a set of exactly specified parameterised distributions that is representative of the uncertainty in the original specification, but also that from this approach we can generate a set of samples that cover the range of possibilities described in the original source in a representative way. When aggregated these samples recover the original uncertainty with a reasonable degree of fidelity. This method is used in our approach to meta-analysis of quantities such as the serial interval that are reported in the literature as uncertainly defined parameter distributions, rather than as single dimensional effect sizes.

References

1. Gelman A. Prior distributions for variance parameters in hierarchical models (comment on article by Browne and Draper). Bayesian Analysis [Internet]. 2006 Sep [cited 2021 Jul 5];1(3):515–34. Available from: https://projecteuclid.org/journals/bayesian-analysis/volume-1/issue-3/Prior-distributions-for-variance-parameters-in-hierarchical-models-comment-on/10.1214/06-BA117A.full
2. Sampling Distribution of Sample Variance | STAT 414 [Internet]. PennState: Statistics Online Courses. [cited 2021 Jul 3]. Available from: https://online.stat.psu.edu/stat414/lesson/26/26.3
3. Nakagami M. The m-Distribution—A General Formula of Intensity Distribution of Rapid Fading. In: Hoffman WC, editor. Statistical Methods in Radio Wave Propagation [Internet]. Pergamon; 1960 [cited 2021 Jul 6]. p. 3–36. Available from: https://www.sciencedirect.com/science/article/pii/B9780080093062500054
4. Douillet PL. Sampling distribution of the variance. In: Proceedings of the 2009 Winter Simulation Conference (WSC) [Internet]. Austin, TX, USA: IEEE; 2009 [cited 2021 Jun 29]. p. 403–14. Available from: http://ieeexplore.ieee.org/document/5429351/

5. O'Neill B. Some Useful Moment Results in Sampling Problems. The American Statistician [Internet]. 2014 Oct 2 [cited 2021 Jul 3];68(4):282–96. Available from: https://doi.org/10.1080/00031305.2014.966589

Serial interval source citations

Bi et al. 2020 Bi, Q. et al. Epidemiology and transmission of COVID-19 in 391 cases and 1286 of their close contacts in Shenzhen, China: a retrospective cohort study. The Lancet Infectious Diseases 20, 911–919 (2020).

Cereda et al. 2020 Cereda, D. et al. The early phase of the COVID-19 outbreak in Lombardy, Italy. arXiv:2003.09320 [q-bio] (2020).

Du et al. 2020 Du, Z. et al. Serial Interval of COVID-19 among Publicly Reported Confirmed Cases. Emerg Infect Dis 26, 1341–1343 (2020).

Kwok et al. 2020 Kwok, K. O., Wong, V. W. Y., Wei, W. I., Wong, S. Y. S. & Tang, J. W.-T. Epidemiological characteristics of the first 53 laboratory-confirmed cases of COVID-19 epidemic in Hong Kong, 13 February 2020. Eurosurveillance 25, 2000155 (2020).

Nishiura et al. 2020 Nishiura, H., Linton, N. M. & Akhmetzhanov, A. R. Serial interval of novel coronavirus (COVID-19) infections. Int. J. Infect. Dis. 93, 284–286 (2020).

Son et al. 2020 Son, H. et al. Epidemiological characteristics of and containment measures for COVID-19 in Busan, Korea. Epidemiol Health 42, (2020).

Zhang et al. 2020 Zhang, J. et al. Evolving epidemiology and transmission dynamics of coronavirus disease 2019 outside Hubei province, China: a descriptive and modelling study. The Lancet Infectious Diseases 20, 793–802 (2020).

Zhao et al. 2020 Zhao, S. et al. Preliminary estimation of the basic reproduction number of novel coronavirus (2019-nCoV) in China, from 2019 to 2020: A data-driven analysis in the early phase of the outbreak. Int. J. Infect. Dis. 92, 214–217 (2020).
